# Violence and depression among men who have sex with men in Tanzania

**DOI:** 10.1186/s12888-017-1456-2

**Published:** 2017-08-15

**Authors:** Lucy R. Mgopa, Jessie Mbwambo, Samuel Likindikoki, Pedro Pallangyo

**Affiliations:** 1 Department of Psychiatry and Mental Health, Muhimbili University of Health and Allied Sciences, School of Medicine, P.O. Box 65001, Dar es Salaam, Tanzania; 2 The Jakaya Kikwete Cardiac Institute, Directorate of Research & Publications, P.O Box 65141, Dar es Salaam, Tanzania

## Abstract

**Background:**

Men who have sex with men (MSM) continue to be at an increased risk of Violence, HIV transmission and Mental Disorders such as depression on top of many other bio-psycho-socio challenges they face as a result of their sexual orientation.

**Methods:**

We recruited 345 MSM using a respondent driven sampling technique. Revised Conflict Tactic Scale, PHQ-9 and questions adapted from the TDHS 2010 were used to assess for violence, depression and HIV-risk behaviors respectively. Continuous and categorical variables were analyzed with student’s t-test and chi-square test respectively. Logistic regression analyses were performed to assess for predictors of depression and HIV-risk behaviors. All tests were two sided and *p* < 0.05 was taken as significance level.

**Results:**

Overall, 325 (94.2%) of participants experienced any form of violence, with emotional violence constituting the majority (90.1%), while physical and sexual violence were reported by 254 (73.6%) and 250 (72.5%) of participants respectively. Depressive symptoms were present in 245 (70.0%) and participants who experienced violence had a 3 times increased risk of depressive symptoms compared to their violence-free counterparts, *p* < 0.001. On the other hand, participants who experienced any form of violence displayed an over 11 times increased rate of depressive symptoms compared to their counterparts who were violence free, *p* < 0.001. Violence experience was found to be the strongest associated factor for depressive symptoms.

**Conclusions:**

The rates of violence, depressive symptoms and HIV risk behaviors amongst MSM are astoundingly high thus necessitating extensive interventions. In view of this, deliberate measures to deal with the reported high rates necessitate joint intervention efforts from the policy makers, health providers and community at large.

**Electronic supplementary material:**

The online version of this article (doi:10.1186/s12888-017-1456-2) contains supplementary material, which is available to authorized users.

## Background

Globally, key populations including MSM are disproportionately affected by violence with resultant physical injuries, mental effects and other health related problems [1].The rates of violence and depression are reportedly higher amongst MSM compared to the general population. Several studies focusing on intimate partner violence (IPV) have reported rates of sexual violence among MSM ranging from 3.2%-18.5%, physical violence 19.2%-22.3% and emotional violence 18.5%-33.1% [2–8]. On the other hand, relatively fewer studies have focused on non-IPV violence and comparatively the rates of violence are uniformly higher compared to IPV. However the differences observed in the rates of violence between non-IPV and IPV amongst studies could be explained by the differences in population characteristics (race, age) and methods (assessment tools, sampling procedure). For instance, sexual violence rates of up-to 57%, and physical and emotional violence of up-to 29.6% and 54.6% respectively have been documented in non-IPV violence studies ([9–11]).

In Tanzania, [1] studied 200 young MSM and found that verbal (48.5%) and moral (32.5%) abuses were the most predominant types of abuse among the sample and the sources were predominantly people in the street and neighbors. Moral abuse in this study was defined as having someone discriminating against or humiliating the participant. Sexual abuse (30%) was mostly from partners, and physical violence (29.5%) was largely from people in the street. Participants in the high-violence level group had a significantly greater number of sexual partners, higher depression scores, and higher internalized homonegativity scores. High level violence was defined as a 3 or more yes answers based on the four questions asked based on four types of violence [1]. Research has also shown high rates of depression and other stress related disorders to be common among MSM, based on their vulnerability to almost negligible political benefits and socio-economic deprivation. Several studies [12–15] have documented depression rates among MSM ranging from 17.0% - 61%. A publication by Ahaneku et al. [16] which utilized the same data set as Anderson et al. [1] found a 46.3% prevalence of depression among 205 Tanzanian MSM, such findings are in consonance with other two other studies conducted in South Africa [17, 18].

Based on the paucity of local data regarding MSM in Tanzania, this study aimed to confirm and extend findings on the magnitude and source of violence and enlighten the extent of depressive symptoms among the MSM population. Furthermore, the study aimed to provide baseline information for gender- and human-rights activists, health care workers, policy makers and other stake holders to develop interventions geared to improve health in the MSM sub-population. We hypothesized that MSM experience high rates of violence and depression which are under evaluated and underdiagnosed.

## Methods

### Study design and setting

This study utilized a cross-sectional analytical design. The study was conducted at the Muhimbili Health Information Centre in Dar es Salaam City, Tanzania. The study was conducted for a period of 3 months, from August – October 2014.

### Study population and inclusion criteria

The study involved men who have sex with men in Dar es Salaam. Inclusion criteria were Men who have sex with men aged at least18 years.

### Sample size calculation

Estimated sample size of the study was calculated by using the formula:

$$ \mathrm{N}=\frac{{\mathrm{Z}}^2\mathrm{P}\left(1-\mathrm{P}\right)}{{\mathrm{d}}^2} $$

Where by: N = estimated sample size, Z = confidence level at 95% (standard value of 1.96), P = Prevalence of violence among men who have sex with men 8% [6, 8], d = Margin of error at 3%. Adjusting for non-response, we added 10% of the estimated sample size *n* = 31.4. Therefore: the minimum estimated and obtained sample size was 345.

### Sampling procedures

The study utilized a respondent driven sampling (RDS) technique. This technique is used to study hidden populations; whereby a subset of members of the population is used to increase the sample size by recruitment of similar members of the group. In this research study, five eligible MSM were identified to serve as seeds. Seeds were chosen from the community based on the convenience of the investigator and were reached individually through phone calls. Each seed was tasked to recruit three participants each, the first and subsequent waves of recruits were similarly tasked to bring three participants until the sample size was attained. Although all interviews took place in a health facility, all participants came from the community. Study interviews were conducted by the principal investigator and two trained research assistants. Participants were given 3000 Tanzanian shillings (US$ 1.37) for their participation and 2000 Tanzanian shillings (US$ 0.91) for each recruit they brought to participate in the study.

### Key measures and assessment tools

Violence was measured using the Revised Conflict Tactic Scale (27 questions). The frequency score ranged from 0 = never, 1 = once, 2 = twice, 3 = 3-5 times, and 4 = 6-10 times, 5 = 11-20 times, 6 = more than 20 times. Three types of violence were assessed: physical, emotional and sexual violence. The scale has been validated in Swahili for use in Tanzania [19]. For our study violence score was dichotomized into “No violence (0) and violence (≥1)”.

Symptoms for depression were assessed by PHQ-9 a validated nine item tool based on the DSM IV diagnostic criteria for depression and it has been used in Sub-Saharan Africa and validated in Swahili language. Each question presented the frequency of the depressive symptoms that one has experienced over the past two weeks, scores ranging from 0 (not at all) to 3 (nearly every day). Scores of 1-4 signified minimal depressive symptoms, 5-9 mild depressive symptoms, 10-14 moderate depressive symptoms and scores of >14 entailed a depressive disorder warranting a combination of antidepressants and psychotherapy in the management [20].

### Data management, entry and analysis

SPSS v.20 software was utilized for data entry and analysis. Student’s t-test and chi square test were employed in comparisons of continuous and categorical variables respectively. Bivariate comparison and subsequent linear logistic regression analyses were used to compare participants with experience of violence versus those without it with respect to the primary end point (depressive symptoms). Statistically significant variables maintained in the regression final model underwent stepwise and forward selection procedures. The multivariate models were fitted with baseline covariates associated with depression by bivariate analyses at the <0.05 significance level. Several potential factors known to be associated with depression including age, education, marital status, sexual positioning and violence experience were included in the logistic model. All analyses were two sided and *p* < 0.05 was used to denote significance.

## Results

We enrolled 345 participants whose mean age was 31 ± 7.31, and the age group 26-35 comprised just over 50% of the total sample (Additional files 1 and 2). While 231 (67.0%) of study subjects were single, those cohabiting with either a man or a woman constituted 11.9% and 6.4% of participants respectively. Primary education was the highest level of education attained in 204 (59.1%), and 240 (69.5%) of participants were currently employed. When asked about positioning preference during sex, 230 (66.7%) were insertive (top), 63 (18.3%) were receptive (bottom), and 52(15.1%) were both insertive and receptive (versatile) Table 1.

**Table 1:** Demographic characteristics of study population by MSM typology

Characteristics	Top (230)	Versatile (52)	Bottom (63)
Age	32.46 ± 7.13	29.96 ± 6.86	26.46 ± 6.28
< 26	42 (18.3)	16 (30.8)	32 (50.8)
26 – 35	123 (53.5)	25 (48.1)	26 (41.3)
36 – 45	56 (24.3)	11 (21.1)	4 (6.3)
46 – 55	8 (3.5)	-	1 (1.6)
> 55	1 (0.4)	-	-
Marital status			
Single	145(63.0)	38(73.1)	48 (76.2)
Married	19 (8.3)	-	2 (3.2)
Divorced/widowed/separated	23 (10.0)	4 (7.7)	3 (4.7)
Cohabiting with a man	24 (10.4)	7 (13.5)	10 (15.9)
Cohabiting with a woman	19 (8.3)	3 (5.8)	-
Education			
At most primary education	158 (68.7)	22 (42.3)	37 (58.7)
Secondary education	66 (28.7)	27 (51.9)	24 (38.1)
Post-secondary education	6 (2.6)	3 (5.8)	2 (3.2)
Occupation			
Unemployed	53 (23.0)	21 (40.4)	26 (41.3)
Employed	175 (76.1)	30 (57.7)	35 (55.5)
Students/retired	2 (0.9)	1 (1.9)	2 (3.2)

Overall, 325 (94.2%) of participants ever experienced any form of violence while 311 (90.1%) had experienced any form of violence within the past 12 months. Emotional, physical and sexual violence were ever experienced by 311 (90.1%), 254 (73.6%) and 250 (72.5%) of participants respectively and experienced by 283(82%), 229 (66.4%) and 225 (65.2%) respectively within the past 12 months. In the assessment of violence within the past 12 months; sexual partners 221 (64.1%) and friends 214 (62.0%) were the most frequent reported perpetrators, others included relatives 180 (52.2%), community members 163 (47.2%), neighbours 118 (34.2%) and police 67 (19.4%), Table 2. With regards to perpetrators and associated type of violence; sexual partners were associated with a majority of sexual violence (66%), police were associated with a majority of physical violence (56%) while friends, relatives, neighbours and community members contributed to a majority of emotional violence, Table 2.

**Table 2:** Frequency of violence perpetrators among MSM who experienced violence

PERPETRATORS	N	%	Emotional	Physical	Sexual
Sexual Partners	221	64.1	99 (45%)	59 (27%)	145 (66%)
Friends	214	62	166 (78%)	44 (21%)	8 (4%)
Relatives	180	52.2	149 (83%)	61 (34%)	1 (0.6%)
Community Members	163	47.2	148 (91%)	65 (40%)	16 (10%)
Neighbours	118	34.2	104 (88%)	21 (18%)	2 (1.7%)
Police	67	19.4	41 (61%)	37 (56%)	1 (1.5%)

Generally, study participants found to have depressive symptoms were 245 (70.01%). Participants who experienced any form of violence displayed an over 11 times increased rate of depressive symptoms compared to their counterparts who were violence free (OR = 11.5, 95% CI 3.7-35.3, *p* < 0.001), Table 3.

**Table 3:** Factors associated with depressive symptoms among MSM

Characteristic	Comparison group	OR	95% CI	*P* Value
Age < 35	Age ≥ 35	1.2	0.7- 1.9	0.60
At most primary education	Post- primary education	0.7	0.5- 1.2	0.21
Single	Married heterosexually	1.3	0.5- 3.2	0.65
Cohabiting with a man		1.4	0.4- 4.3	0.59
Versatile	Top	1.5	0.8- 3.1	0.24
Bottom		0.8	0.4- 1.4	0.37
Experienced violence	Not experienced violence	11.5	3.7- 15.3	<0.001

## Discussion

### Magnitude and type of violence

In this present study, we found the prevalence of any form of violence amongst MSM to be 94.2%. Even after categorizing violence by type i.e. physical, emotional and sexual, still each form was experienced by at least three quarters of participants. A study done by Chellan et al. [10] revealed a prevalence of 29.6%, 54.6%, 57% in physical, emotional and sexual forms of non-IPV violence respectively. Another study by [11] in India reported non-IPV sexual violence rates of 18%. A local study by [1] revealed non-IPV rates of physical violence of 29.5%, sexual violence 30%, and emotional violence 48.5%. In the recent times, MSM living in Tanzania has faced even more hostility than ever before. Such hostility includes, arrest, persecution, discrimination, harassments, alienation from family and faith, lack of access to social services [21, 22]. These factors in particular amongst others could offer the explanation as to why the rates of violence observed in this study are significantly higher than the ones observed in the previous published works.

Nearly two thirds of study participants reported their sexual partners as the most common perpetrators, while relatives and friends were reported by over half of MSM. Like other studies [1] it was observed that confidants who under natural circumstances are expected to be the source of hope and strength were the leading perpetrators. Assessment of factors associated with violence in a 6-item ‘age, education, marital status, depressive symptoms, and bottom and versatile sexual positioning’ logistic regression model revealed depressive symptoms were the strongest associated factor.

With respect to burden of violence and depression, our findings echo those of Anderson et al. [1]. However, the magnitude from this current study are appreciably higher compared to Anderson’s [1] data. This difference could probably be explained by the fact that participants in this current study were older than the ones in Anderson’s study and potentially had more years of exposure to violence and depression. Our findings potentially reflect the fact that MSM face more hostility currently than a few years ago. Nevertheless, the rates of depression among MSM remain considerably high, displaying a strong association with violence.

### Prevalence and associated factors of depressive symptoms

Nearly three quarters of our participants displayed depressive symptoms, with over 90% of these falling in the category of minimal to mild depression. Subjects with violence experience had over 11 times odds of having depressive symptoms compared to violence free participants, (OR 11.5, 95 CI 3.5-48.1, *p* < 0.001). Studies conducted (by [23, 12, 13, 24, 25, 15]) revealed significantly lower depression rates (18%-61%) than the present study. Violence was found to be the strongest associated factor of depressive symptoms in this study. Other potential factors including age, education, marital status and typology didn’t prove to be significant factors of depression in this set up. A study by Klein et al., [24] produced similar results as far as violence is concerned, however, in contrary to our study, low education was a shared predictor in the (Klein et al., [24] and Hirshfield et al. [13] studies, while marriage to a woman and single status were predictors in the Hirshfield et al. [13] study alone. Being MSM in this setting and similar continues to be a reason for extensive discrimination and abuse; as a result men fail to seek for medical-legal help as expected. This inflames distress in an already distressed population full of fear of disclosing their sexual orientation and depression is inevitably high. This not only complicates the lives of MSM but also does hinder both mental health seeking behaviour and service provision [26, 27].

### Limitations

Due to the observational nature of this study our findings could have potentially be exposed to interviewers’ bias and recall bias. However, all interviewers were qualified personnel working in the department of psychiatry and mental health but also underwent a thorough training to familiarize themselves with study objectives and questionnaire. Although we are not in position to infer causality, our current understanding however is that both violence and depression are strongly interlinked each with a potential to cause or result from one another.

## Conclusions

The rate of violence and depressive symptoms amongst MSM in Tanzania is very high. In view of this, clinicians should strive to provide standard care and service regardless of individual’s sexual orientation. Policy makers need to design policies and programs aiming at preventing abuse and reducing stigma through safeguarding of human rights of MSM. Furthermore, studies are needed to identify the non-MSM community’s knowledge, attitude and practices towards MSM in this setting, such information is vital in designing interventions tailored specifically to reduce violence and depression.

## Additional files

Additional file 1: My data set. (XLS 504 kb)

Additional file 2: Variable. (DOCX 21 kb)
