# Supplementary material for: Violence and depression among men who have sex with men in Tanzania
Source: BMC Psychiatry. 2017 Aug 15;17:296. doi: 10.1186/s12888-017-1456-2 (PMC5558659; doi:10.1186/s12888-017-1456-2)
Supplement: Supplementary file 2 — Variable. (DOCX 21 kb) [file 12888_2017_1456_MOESM2_ESM.docx]

| **Variable Name** | **Variable description** |
| --- | --- |
| Ageofparticipant | Age of participant in years |
| Maritalstatus | Marital Status:  1. Single  2. Married  3. Separated/divorced  4. Widowed  5. Cohabiting with a man  6.Cohabiting with a woman |
| Areaofresident | Area of residence based on districts: Ilala, Kinondoni and Temeke |
| LevelofEducation | Level of education:  1. No Formal education  2. Primary Education  3. Secondary Education  4. Some college education  5. University Graduate |
| Religion | Religion:  1. Muslim  2. Christian  3. Pagan |
| Occupationalstatus | Occupational status:  1. Unemployed  2. Employed  3. Self employed  4. Student  5. Retired |
| TypeofMSM | Type of MSM (men who have sex with men)  1. Top  2. Versatile  3. Bottom |
| Someoneshowedcareformeeventhoughwedisagreed | Someone showed care for me even though we disagreed (None Tactic –ever experienced):  0. This never happened  1 = Happened once  2 = 2 times in the past  3 = 3-5 times in the past  4 = 6-10 times in the past  5= 11-20 times in the past  6= more than 20 times in the past |
| Someoneexplainedhissideofdisagreementtome | Someone explained his side of disagreement to me(none tactic- ever experienced)  0. This never happened  1 = Happened once  2 = 2 times in the past  3 = 3-5 times in the past  4 = 6-10 times in the past  5= 11-20 times in the past  6= more than 20 times in the past |
| Someonesworeorinsultedme | Someone swore or insulted me (emotional violence- ever experienced)  0. This never happened  1 = Happened once  2 = 2 times in the past  3 = 3-5 times in the past  4 = 6-10 times in the past  5= 11-20 times in the past  6= more than 20 times in the past |
| Someoneyelledorshoutedatme | Someone yelled or shouted at me (emotional violence- ever experienced)  0. This never happened  1 = Happened once  2 = 2 times in the past  3 = 3-5 times in the past  4 = 6-10 times in the past  5= 11-20 times in the past  6= more than 20 times in the past |
| Someonestompedoutofthehouseduringa disagreement | Someone stomped out of the house during a disagreement (emotional violence- ever experienced)  0. This never happened  1 = Happened once  2 = 2 times in the past  3 = 3-5 times in the past  4 = 6-10 times in the past  5= 11-20 times in the past  6= more than 20 times in the past |
| Someonedidsomethingtospiteme | Someone did something to spite me (emotional violence- ever experienced)  0. This never happened  1 = Happened once  2 = 2 times in the past  3 = 3-5 times in the past  4 = 6-10 times in the past  5= 11-20 times in the past  6= more than 20 times in the past |
| Someoneshowedrespectformyfeelingsaboutanissue | Someone showed respect for my feelings about an issue (none tactic- ever experienced)  0. This never happened  1 = Happened once  2 = 2 times in the past  3 = 3-5 times in the past  4 = 6-10 times in the past  5= 11-20 times in the past  6= more than 20 times in the past |
| Someonedestroyedsomethingthatbelongedtome | Someone destroyed something that belonged to me (emotional violence-ever experienced)  0. This never happened  1 = Happened once  2 = 2 times in the past  3 = 3-5 times in the past  4 = 6-10 times in the past  5= 11-20 times in the past  6= more than 20 times in the past |
| Someonethreatenedtohitorthrowsomethingatme | Someone threatened to hit or throw something at me (emotional violence-ever experienced)  0. This never happened  1 = Happened once  2 = 2 times in the past  3 = 3-5 times in the past  4 = 6-10 times in the past  5= 11-20 times in the past  6= more than 20 times in the past |
| Someonethrewsomethingatmethatcouldhurt | Someone threw something at me that could hurt (physical violence- ever experienced)  0. This never happened  1 = Happened once  2 = 2 times in the past  3 = 3-5 times in the past  4 = 6-10 times in the past  5= 11-20 times in the past  6= more than 20 times in the past |
| Someonetwistedmyarmormyhair | Someone twisted my arm or my hair (physical violence- ever experienced)  0. This never happened  1 = Happened once  2 = 2 times in the past  3 = 3-5 times in the past  4 = 6-10 times in the past  5= 11-20 times in the past  6= more than 20 times in the past |
| Someoneshovedorpushedme | Someone shoved or pushed me (physical violence- ever experienced)  0. This never happened  1 = Happened once  2 = 2 times in the past  3 = 3-5 times in the past  4 = 6-10 times in the past  5= 11-20 times in the past  6= more than 20 times in the past |
| Someonebeatmeup | Someone beat me up (physical violence- ever experienced)  0. This never happened  1 = Happened once  2 = 2 times in the past  3 = 3-5 times in the past  4 = 6-10 times in the past  5= 11-20 times in the past  6= more than 20 times in the past |
| Someonegrabbedme | Someone grabbed me (physical violence- ever experienced)  0. This never happened  1 = Happened once  2 = 2 times in the past  3 = 3-5 times in the past  4 = 6-10 times in the past  5= 11-20 times in the past  6= more than 20 times in the past |
| Someonethreatenedmewithaknifeoragun | Someone threatened me with a knife or a gun (emotional violence-ever experienced)  0. This never happened  1 = Happened once  2 = 2 times in the past  3 = 3-5 times in the past  4 = 6-10 times in the past  5= 11-20 times in the past  6= more than 20 times in the past |
| Someoneusedaknifeoragunonme | Someone used a knife or a gun on me (Physical violence-ever experienced)  0. This never happened  1 = Happened once  2 = 2 times in the past  3 = 3-5 times in the past  4 = 6-10 times in the past  5= 11-20 times in the past  6= more than 20 times in the past |
| Someonechokedme | Someone choked me (Physical violence-ever experienced)  0. This never happened  1 = Happened once  2 = 2 times in the past  3 = 3-5 times in the past  4 = 6-10 times in the past  5= 11-20 times in the past  6= more than 20 times in the past |
| Someonepunchedmeorhitmewithsomethingthat couldhurt | Someone punched me or hit me with something that could hurt (physical violence-ever experienced)  0. This never happened  1 = Happened once  2 = 2 times in the past  3 = 3-5 times in the past  4 = 6-10 times in the past  5= 11-20 times in the past  6= more than 20 times in the past |
| Someoneburnedorscaldedmeonpurpose | Someone burned or scalded me on purpose (Physical violence-ever experienced)  0. This never happened  1 = Happened once  2 = 2 times in the past  3 = 3-5 times in the past  4 = 6-10 times in the past  5= 11-20 times in the past  6= more than 20 times in the past |
| Someonesuggestedacompromisetoadisagreement | Someone suggested a compromise to a disagreement (none tactic- ever experienced)  0. This never happened  1 = Happened once  2 = 2 times in the past  3 = 3-5 times in the past  4 = 6-10 times in the past  5= 11-20 times in the past  6= more than 20 times in the past |
| Someonekickedme | Someone kicked me (physical violence- ever experienced)  0. This never happened  1 = Happened once  2 = 2 times in the past  3 = 3-5 times in the past  4 = 6-10 times in the past  5= 11-20 times in the past  6= more than 20 times in the past |
| Someonemademehavesexwithoutacondom | Someone made me have sex without a condom (sexual violence-ever experienced)  0. This never happened  1 = Happened once  2 = 2 times in the past  3 = 3-5 times in the past  4 = 6-10 times in the past  5= 11-20 times in the past  6= more than 20 times in the past |
| Someoneusedforcetomakemehavesex | Someone used force to make me have sex (sexual violence-ever experienced)  0. This never happened  1 = Happened once  2 = 2 times in the past  3 = 3-5 times in the past  4 = 6-10 times in the past  5= 11-20 times in the past  6= more than 20 times in the past |
| Someoneusedforcetomakemehaveoralsex | Someone used force to make me have oral sex (sexual violence-ever experienced)  0. This never happened  1 = Happened once  2 = 2 times in the past  3 = 3-5 times in the past  4 = 6-10 times in the past  5= 11-20 times in the past  6= more than 20 times in the past |
| SomeoneinsistedonsexwhenIdidnotwantto(butdidnot usephysicalforce) | Someone insisted on sex when I did not want to (but did not use physical force) (sexual violence-ever experienced)  0. This never happened  1 = Happened once  2 = 2 times in the past  3 = 3-5 times in the past  4 = 6-10 times in the past  5= 11-20 times in the past  6= more than 20 times in the past |
| Someoneusedthreatstomakemehaveoralsex | Someone used threats to make me have oral sex (sexual violence-ever experienced)  0. This never happened  1 = Happened once  2 = 2 times in the past  3 = 3-5 times in the past  4 = 6-10 times in the past  5= 11-20 times in the past  6= more than 20 times in the past |
| Someoneusedthreatstomakemehavesex | Someone used threats to make me have sex (sexual violence-ever experienced)  0. This never happened  1 = Happened once  2 = 2 times in the past  3 = 3-5 times in the past  4 = 6-10 times in the past  5= 11-20 times in the past  6= more than 20 times in the past |
| Someoneshowedcareformeeventhoughwedisagreed2 | Someone showed care for me even though we disagreed (None Tactic –past 12 months):  0. This never happened  1 = Happened once  2 = 2 times in the past  3 = 3-5 times in the past  4 = 6-10 times in the past  5= 11-20 times in the past  6= more than 20 times in the past |
| Someoneexplainedhissideofdisagreementtome2 | Someone explained his side of disagreement to me(none tactic- past 12 months)  0. This never happened  1 = Happened once  2 = 2 times in the past  3 = 3-5 times in the past  4 = 6-10 times in the past  5= 11-20 times in the past  6= more than 20 times in the past |
| Someonesworeorinsultedme2 | Someone swore or insulted me (emotional violence- past 12 months)  0. This never happened  1 = Happened once  2 = 2 times in the past  3 = 3-5 times in the past  4 = 6-10 times in the past  5= 11-20 times in the past  6= more than 20 times in the past |
| Someoneyelledorshoutedatme2 | Someone yelled or shouted at me (emotional violence- past 12 months)  0. This never happened  1 = Happened once  2 = 2 times in the past  3 = 3-5 times in the past  4 = 6-10 times in the past  5= 11-20 times in the past  6= more than 20 times in the past |
| Someonestompedoutofthehouseduringa disagreement2 | Someone stomped out of the house during a disagreement (emotional violence- past 12 months)  0. This never happened  1 = Happened once  2 = 2 times in the past  3 = 3-5 times in the past  4 = 6-10 times in the past  5= 11-20 times in the past  6= more than 20 times in the past |
| Someonedidsomethingtospiteme2 | Someone did something to spite me (emotional violence- past 12 months)  0. This never happened  1 = Happened once  2 = 2 times in the past  3 = 3-5 times in the past  4 = 6-10 times in the past  5= 11-20 times in the past  6= more than 20 times in the past |
| Someoneshowedrespectformyfeelingsaboutanissue2 | Someone showed respect for my feelings about an issue (none tactic- past 12 months)  0. This never happened  1 = Happened once  2 = 2 times in the past  3 = 3-5 times in the past  4 = 6-10 times in the past  5= 11-20 times in the past  6= more than 20 times in the past |
| Someonedestroyedsomethingthatbelongedtome2 | Someone destroyed something that belonged to me (emotional violence- past 12 months)  0. This never happened  1 = Happened once  2 = 2 times in the past  3 = 3-5 times in the past  4 = 6-10 times in the past  5= 11-20 times in the past  6= more than 20 times in the past |
| Someonethreatenedtohitorthrowsomethingatme2 | Someone threatened to hit or throw something at me (emotional violence- past 12 months)  0. This never happened  1 = Happened once  2 = 2 times in the past  3 = 3-5 times in the past  4 = 6-10 times in the past  5= 11-20 times in the past  6= more than 20 times in the past |
| Someonethrewsomethingatmethatcouldhurt2 | Someone threw something at me that could hurt (physical violence-past 12 months)  0. This never happened  1 = Happened once  2 = 2 times in the past  3 = 3-5 times in the past  4 = 6-10 times in the past  5= 11-20 times in the past  6= more than 20 times in the past |
| Someonetwistedmyarmormyhair2 | Someone twisted my arm or my hair (physical violence- past 12 months)  0. This never happened  1 = Happened once  2 = 2 times in the past  3 = 3-5 times in the past  4 = 6-10 times in the past  5= 11-20 times in the past  6= more than 20 times in the past |
| Someoneshovedorpushedme2 | Someone shoved or pushed me (physical violence- past 12 months)  0. This never happened  1 = Happened once  2 = 2 times in the past  3 = 3-5 times in the past  4 = 6-10 times in the past  5= 11-20 times in the past  6= more than 20 times in the past |
| Someonebeatmeup2 | Someone beat me up (physical violence- past 12 months)  0. This never happened  1 = Happened once  2 = 2 times in the past  3 = 3-5 times in the past  4 = 6-10 times in the past  5= 11-20 times in the past  6= more than 20 times in the past |
| Someonegrabbedme2 | Someone grabbed me (physical violence- past 12 months)  0. This never happened  1 = Happened once  2 = 2 times in the past  3 = 3-5 times in the past  4 = 6-10 times in the past  5= 11-20 times in the past  6= more than 20 times in the past |
| Someonethreatenedmewithaknifeoragun2 | Someone threatened me with a knife or a gun (emotional violence-past 12 months)  0. This never happened  1 = Happened once  2 = 2 times in the past  3 = 3-5 times in the past  4 = 6-10 times in the past  5= 11-20 times in the past  6= more than 20 times in the past |
| Someoneusedaknifeoragunonme2 | Someone used a knife or a gun (physical violence-past 12 months)  0. This never happened  1 = Happened once  2 = 2 times in the past  3 = 3-5 times in the past  4 = 6-10 times in the past  5= 11-20 times in the past  6= more than 20 times in the past |
| Someonechokedme2 | Someone choked me (physical violence- past 12 months)  0. This never happened  1 = Happened once  2 = 2 times in the past  3 = 3-5 times in the past  4 = 6-10 times in the past  5= 11-20 times in the past  6= more than 20 times in the past |
| Someonepunchedmeorhitmewithsomethingthat couldhurt2 | Someone punched me or hit me with something that could hurt (physical violence-past 12 months)  0. This never happened  1 = Happened once  2 = 2 times in the past  3 = 3-5 times in the past  4 = 6-10 times in the past  5= 11-20 times in the past  6= more than 20 times in the past |
| Someoneburnedorscaldedmeonpurpose2 | Someone burned or scalded me on purpose (Physical violence-past 12 months)  0. This never happened  1 = Happened once  2 = 2 times in the past  3 = 3-5 times in the past  4 = 6-10 times in the past  5= 11-20 times in the past  6= more than 20 times in the past |
| Someonesuggestedacompromisetoadisagreement2 | Someone suggested a compromise to a disagreement (none tactic- past 12 months)  0. This never happened  1 = Happened once  2 = 2 times in the past  3 = 3-5 times in the past  4 = 6-10 times in the past  5= 11-20 times in the past  6= more than 20 times in the past |
| Someonekickedme2 | Someone kicked me (physical violence- past 12 months)  0. This never happened  1 = Happened once  2 = 2 times in the past  3 = 3-5 times in the past  4 = 6-10 times in the past  5= 11-20 times in the past  6= more than 20 times in the past |
| Someonemademehavesexwithoutacondom2 | Someone made me have sex without a condom (sexual violence-past 12 months)  0. This never happened  1 = Happened once  2 = 2 times in the past  3 = 3-5 times in the past  4 = 6-10 times in the past  5= 11-20 times in the past  6= more than 20 times in the past |
| Someoneusedforcetomakemehavesex2 | Someone used force to make me have sex(sexual violence-past 12 months)  0. This never happened  1 = Happened once  2 = 2 times in the past  3 = 3-5 times in the past  4 = 6-10 times in the past  5= 11-20 times in the past  6= more than 20 times in the past |
| Someoneusedforcetomakemehaveoralsex2 | Someone used force to make me have oral sex (sexual violence-past 12 months)  0. This never happened  1 = Happened once  2 = 2 times in the past  3 = 3-5 times in the past  4 = 6-10 times in the past  5= 11-20 times in the past  6= more than 20 times in the past |
| SomeoneinsistedonsexwhenIdidnotwantto(butdidnot usephysicalforce) | Someone insisted on sex when I did not want to(but did not use physical force(sexual violence- past 12 months)  0. This never happened  1 = Happened once  2 = 2 times in the past  3 = 3-5 times in the past  4 = 6-10 times in the past  5= 11-20 times in the past  6= more than 20 times in the past |
| Someoneusedthreatstomakemehaveoralsex2 | Someone used threats to make me have oral sex (sexual violence-past 12 months)  0. This never happened  1 = Happened once  2 = 2 times in the past  3 = 3-5 times in the past  4 = 6-10 times in the past  5= 11-20 times in the past  6= more than 20 times in the past |
| Someoneusedthreatstomakemehavesex2 | Someone used threats to make me have sex (sexual violence- past 12 months)  0. This never happened  1 = Happened once  2 = 2 times in the past  3 = 3-5 times in the past  4 = 6-10 times in the past  5= 11-20 times in the past  6= more than 20 times in the past |
| Partner | Perpetrator partner:  1. Yes  2. No |
| Relative | Perpetrator Relative:  1. Yes  2. No |
| Friend | Perpetrator Friend:  1. Yes  2. No |
| Neighbour | Perpetrator Neighbour:  1. Yes  2. No |
| Police | Perpetrator Police:  1. Yes  2. No |
| Community Member | Perpetrator Community Member:  1. Yes  2. No |
| Littleinterestorpleasureindoingthings | Little interest or pleasure in doing things:  0. Not at all  1. Several days  2. More half of the days  3. Nearly everyday |
| Feelingdowndepressedhopeless | Feeling down, depressed or hopeless:  0. Not at all  1. Several days  2. More half of the days  3. Nearly everyday |
| Troublefallingstayingasleeporsleepingtoomuch | Trouble falling, staying asleep or sleeping too much  0. Not at all  1. Several days  2. More half of the days  3. Nearly everyday |
| Feelingtiredorhavinglittleenergy | Feeling tired or having little energy  0. Not at all  1. Several days  2.More half of the days  3.Nearly everyday |
| Poorappetiteorovereating | Poor appetite or overeating  0. Not at all  1. Several days  2.More half of the days  3.Nearly everyday |
| Feelingbadaboutyourselforthatyouareafailure | Feeling bad about yourself or that you are a failure  0. Not at all  1. Several days  2.More half of the days  3.Nearly everyday |
| Troubleconcentratingonthingssuchasreadingorwatching | Trouble concentrating on things such as reading or watching  0. Not at all  1. Several days  2.More half of the days  3.Nearly everyday |
| Movingorspeakingsoslowlythatotherpeoplecouldhavenoticed | Moving or speaking so slowly that other people could have noticed  0. Not at all  1. Several days  2.More half of the days  3.Nearly everyday |
| Thoughtsthatyouwouldbebetteroffdeadorofhurtingyourself | Thoughts that you would be better off dead or hurting yourself  0. Not at all  1. Several days  2.More half of the days  3.Nearly everyday |
| Total | Total |
| Howdifficulthavetheseproblemsmadeitforyoutodoyourwork | How difficult have these problems made it for you to do your work, take care of things at home or get along with people  1. Not difficult at all  2. Somewhat difficult  3. Very difficult  4. Extremely Difficult |
|  |  |
